# Spasmodic Wry-Neck and Other Spasmodic Affections of the Head, Face, and Neck

**Published:** 1892-03

**Authors:** 


*Spasmodic Wry-neck and other Spasmodic Affections of the Head, Face, and Neck.* By NOBLE SMITH, F.R.C.S.E. Pp. 55. London: Smith, Elder & Co. 1891.

The true form of spasmodic wry-neck as distinguished from the hysterical is well known to follow a very obstinate course, and to be little amenable to treatment. In the failure of therapeutic remedies, various surgical procedures have been proposed and carried out from time to time, hitherto without much success. The latest operation, which Mr. Noble Smith advocates in this pamphlet, is the excision of a portion of the spinal accessory just before it enters the sterno-mastoid, and if the muscles at the back are affected, of portions of the posterior cervical nerves. This operation has now been adopted in several obstinate cases with gratifying results, and would seem to promise success in the future.

